# Knockdown of HPRT for Selection of Genetically Modified Human Hematopoietic Progenitor Cells

**DOI:** 10.1371/journal.pone.0059594

**Published:** 2013-03-15

**Authors:** Rashmi Choudhary, Dmitry Baturin, Susan Fosmire, Brian Freed, Christopher C. Porter

**Affiliations:** 1 Department of Pediatrics, University of Colorado School of Medicine, Aurora, Colorado, United States of America; 2 Department of Medicine, University of Colorado School of Medicine, Aurora, Colorado, United States of America; 3 Department of Immunology, University of Colorado School of Medicine, Aurora, Colorado, United States of America; Emory University School of Medicine, United States of America

## Abstract

The inability to obtain sufficient numbers of transduced cells remains a limitation in gene therapy. One strategy to address this limitation is *in vivo* pharmacologic selection of transduced cells. We have previously shown that knockdown of HPRT using lentiviral delivered shRNA facilitates efficient selection of transduced murine hematopoietic progenitor cells (HPC) using 6-thioguanine (6TG). Herein, we now extend these studies to human HPC. We tested multiple shRNA constructs in human derived cell lines and identified the optimal shRNA sequence for knockdown of HPRT and 6TG resistance. We then tested this vector in human umbilical cord blood derived HPC *in vitro* and in NOD/SCID recipients. Knockdown of HPRT effectively provided resistance to 6TG *in vitro*. 6TG treatment of mice resulted in increased percentages of transduced human CD45^+^ cells in the peripheral blood and in the spleen in particular, in both myeloid and lymphoid compartments. 6TG treatment of secondary recipients resulted in higher percentages of transduced human cells in the bone marrow, confirming selection from the progeny of long-term repopulating HPCs. However, the extent of selection of cells in the bone marrow at the doses of 6TG tested and the toxicity of higher doses, suggest that this strategy may be limited to selection of more committed progenitor cells. Together, these data suggest that human HPC can be programmed to be resistant to purine analogs, but that HPRT knockdown/6TG-based selection may not be robust enough for *in vivo* selection.

## Introduction

In the past decade, several clinical trials have been carried out that have highlighted both the promise of gene therapy [1], [2], [3], [4] and the potential harm from the uncontrolled integrations of the viral vectors used to deliver the therapeutic transgene [5], [6]. In contrast to gamma-retroviral vectors, which integrate preferentially near transcriptional start sites, lentiviral vectors (LVs) show safer integration profiles and are considered less genotoxic than gamma-retroviral vectors [7], [8]. Lentiviral vectors have been used in clinical trials, however these studies used myeloablative conditioning and/or relatively high multiplicities of infection (MOIs) for transduction in order to achieve sufficient numbers of transduced cells [3], [9], which may increase the risk for insertional mutagenesis. An alternative approach would be to use low MOI for transduction and use a selective agent for increasing the proportion of gene transduced cells, particularly for those diseases in which the transgene does not provide a selective advantage.

To this end, the multidrug resistance gene (MDR-1) [10], [11], [12], [13], the dihydrofolate-reductase gene (DHFR) [14], [15], [16], [17] and the O^6^-methylguanine-DNA-methyltransferase gene (MGMT) [18], [19], [20], [21], [22] have been tested extensively in this context. It has been shown that the use of all 3 systems results in an enrichment of gene-corrected blood cells in mice. However, in most cases, the enrichment was transient, suggesting that more committed HPC with limited self-renewal potential were primarily selected. Selection at the stem cell level could unambiguously be demonstrated only with the MGMT system, resulting in efficient and stable MGMT^P140K^-mediated multi-lineage selection in both macaque and baboon nonhuman primate models [19], and the first report of its clinical testing was recently reported [23]. Furthermore, the carcinogenic potential of alkylating drugs represents a considerable risk for clinical applications of this approach [24], [25].

We have developed an alternative approach to HPC selection that relies on shRNA mediated knockdown of hypoxanthine phosphoribosyl transferase (HPRT), the enzyme required for metabolizing purine analogs like 6-thioguanine (6TG) into active agents [26]. This approach has several advantages. First, the sequence needed to induce drug resistance is very short (48 bases), making subsequent inclusion of a therapeutic gene and regulatory elements simpler and more efficient. Second, 6TG is a purine analog that has been used clinically for decades and is routinely titrated to desired hematopoietic toxicity. And although anti-metabolites may contribute to leukemogenesis [27], in the absence of concomitant administration of highly genotoxic medications, this risk is quite low [27], [28]. Third, this strategy provides drug resistance without the introduction of a mutated protein that might lead to immunologic elimination of transduced cells. More recently, another group showed that 6TG can be used for conditioning as well as highly efficient *in vivo* chemo-selection of HPRT-deficient HSC in mouse models [29]. However, the effectiveness of this strategy in human hematopoietic progenitors has not yet been demonstrated. Herein we demonstrate specific, selective enrichment and expansion of human cell lines and CD34^+^ umbilical cord blood (UCB) cells expressing shRNA targeting HPRT and treated with 6TG *in vitro*. Further, transplanting transduced UCB cells into NOD/SCID mice and treatment with 6TG resulted in maintenance of transduced myeloid and lymphoid progeny in primary as well as secondary recipients, but not robust selection in the bone marrow. Together, these data suggest that human HPC can be programmed to be resistant to purine analogs, but that HPRT knockdown/6TG-based selection may not be sufficient for *in vivo* selection.

## Results

### Knockdown of HPRT Provides Resistance to 6TG in Human Lymphoid and Myeloid Cell Lines

**Figure 1:**
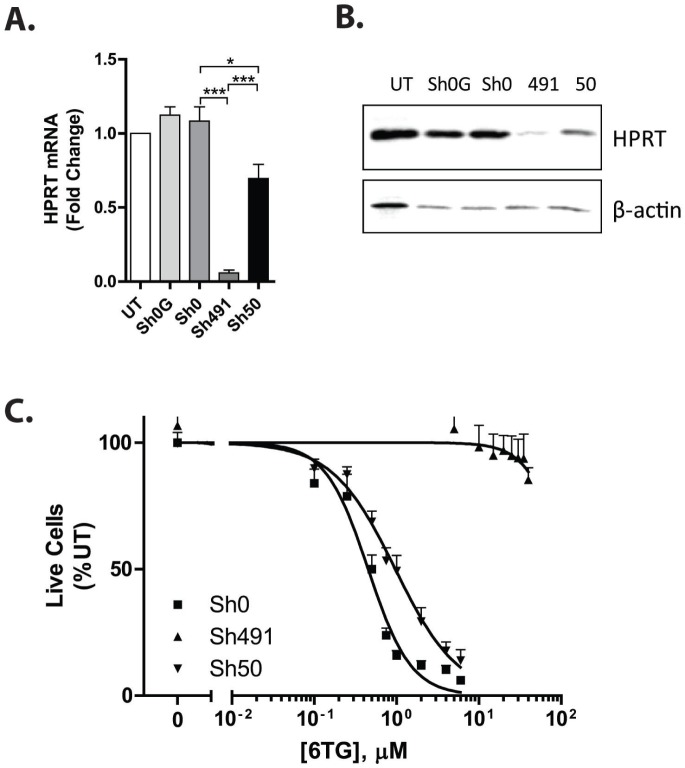
Knockdown of HPRT confers resistance to 6TG in human hematopoietic cells. Molm13 cells were transduced with lentiviral vectors expressing non-silencing control sequences (sh0 and sh0G) or shRNAs directed against HPRT (sh491 and sh50) and selected in puromycin. A & B. Construct 491 most effectively knocks down HPRT. The extent of knockdown of HPRT was measured by reverse-transcription, real-time PCR with primers specific for HPRT (A) and western blotting (B). Constructs 491 and 50 efficiently knocked down HPRT, as compared to untransduced controls. The extent of knockdown was significantly greater with construct 491. C. Construct 491 provides the greatest resistance to 6TG. Transduced cells were treated with increasing doses of 6TG, and the number of live cells was measured by flow cytometry and propidium iodide exclusion. Construct 491 provided the best resistance to 6TG.

We first screened 5 shRNA constructs targeting human HPRT in the human acute lymphoblastic leukemia cell line, REH (See **Supplemental Table 1** for details of shRNA constructs). Of these, construct sh50 provided the best knockdown of HPRT and resistance to treatment with 6TG (**Supplemental** **figure 1**). This construct was then compared to sh491 in subsequent experiments, as sh491 was most effective in knocking down HPRT in murine hematopoietic cells, and its sequence is also complementary to human HPRT. As shown in **Fig. 1a**, compared to cells transduced with non-silencing control shRNAs (sh0 and sh0G), construct sh491 resulted in 90% reduction in HPRT mRNA in human acute myeloid leukemia Molm13 cells as determined by real-time RT-PCR, while that of sh50 was only 50%. Consistent with qPCR results, western blotting of whole cell lysates demonstrated greater knockdown of HPRT protein expression by construct sh491 compared to sh50 (**Fig. 1b**). To determine if knockdown of HPRT would provide resistance to 6TG, transduced Molm13 cells were cultured in the presence or absence of different concentrations of 6TG for 72 h. While treatment with 6TG inhibited proliferation of control transduced cells in a dose dependent fashion, as measured by direct cell counting, cells in which HPRT was knocked down were relatively resistant to 6TG (**Fig. 1c**). Resistance to 6TG was associated with the extent of knockdown of HPRT. Sh491 transduced cells, which showed a greater reduction in HPRT protein and mRNA levels, had the highest IC50 values (114.5 µM [95% confidence interval: 6.9–1911]) and continued to proliferate even at the highest concentrations of 6TG tested, while sh50 transduced cells had lower IC50 values (0.97 µM [0.83–1.1]), albeit greater than sh0 transduced cells (0.45 µM [0.40–0.51]; **Fig. 1c**). Similar results were obtained in another AML cell line, MV4-11 (data not shown) as well as in REH cells (**Supplemental** **Figure 2**). Resistance to 6TG in cells in which HPRT was knocked down was attributed to lower rates of apoptosis upon treatment with 6TG (**Supplemental** **Figure 3a & b**). The extent of knockdown and resistance to 6TG was persistent over the course of multiple population doublings (**Supplemental** **Figure 3c**), indicating that knockdown of HPRT does not provide a proliferative disadvantage in these cell lines *in vitro*. Together these data suggest that knockdown of HPRT with construct 491 can most effectively provide resistance to 6TG in human myeloid and lymphoid derived cells.

**Figure 2:**
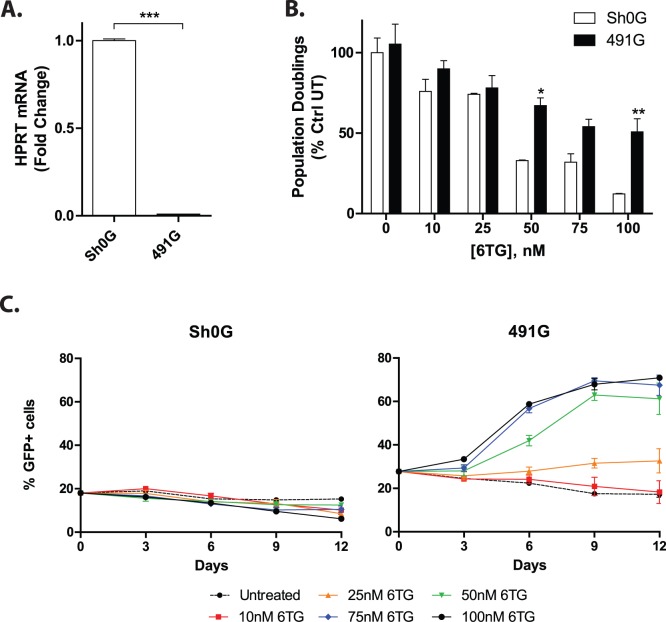
Knockdown of HPRT confers resistance to 6TG in human umbilical cord hematopoietic progenitor cells. A. Construct 491 efficiently depletes HPRT expression in human umbilical cord hematopoietic progenitor cells. CD34^+^ cells isolated from human umbilical cord blood were transduced with vectors expressing GFP and shRNA against HPRT (sh491G) or non-silencing control (sh0G). Cells were sorted for GFP expression, total RNA was isolated and analyzed by reverse transcription, real time PCR. Construct sh491G reduced HPRT expression by 90%. B. Human HPC with reduced HPRT continue to proliferate in 6TG. Cells were transduced as in A, and unsorted cells were cultured in the presence of cytokines, with re-seeding in fresh media and 6TG every 72 hours. Cells were assessed by flow cytometry for GFP, propidium iodide exclusion and counting every 72 hours. The number of population doublings of GFP^+^ cells at day 6 was determined, and is depicted as a percentage of untreated, non-silencing control transduced cells. C. Knockdown of HPRT allows for selection of transduced cells in 6TG. UCB cells were transduced, cultured and treated as in Figure 2B. The percentage of GFP^+^ cells was measured every 72 hours. Note the dose dependent increase in GFP^+^ cells with time in cells transduced with 491G.

**Figure 3:**
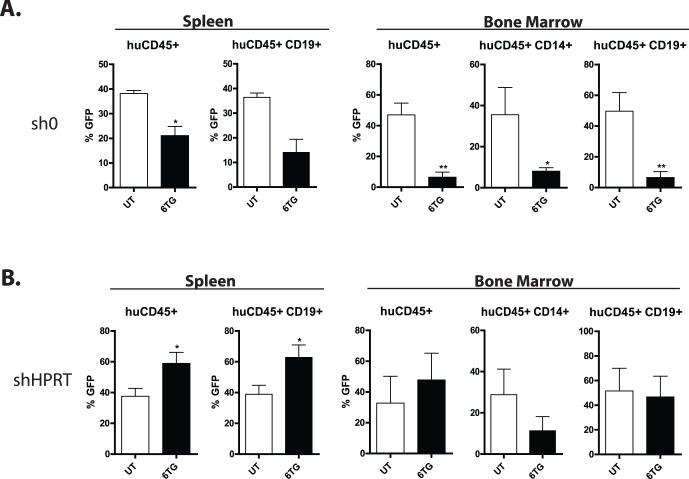
Knockdown of HPRT protects human cells from the toxic effects of 6TG in vivo. Human CD34^+^ umbilical cord blood cells were transduced with GFP expressing vectors with non-silencing shRNA (**A**) or shRNA directed against HPRT (**B**) and transplanted into sub-lethally irradiated recipients. Mice were then treated with 6TG 2 mg/kg/d in the drinking water or left untreated. After 6 weeks, the spleens and bone marrow were analyzed by flow cytometry for cell surface markers for total human leukocytes, B-lymphocyte and myeloid subpopulations, and GFP. A. Cells transduced with non-silencing control were selectively depleted with treatment with 6TG. The percentages of GFP^+^ human leukocytes (huCD45^+^), B-lymphocytes (huCD45^+^CD19^+^), and myeloid cells (huCD45^+^CD14^+^) from the spleen and bone marrow are depicted. There were 2 untreated and 5 6TG treated mice in this experiment. B. Cells transduced with shRNA directed against HPRT are protected from the cytotoxic effects of 6TG. The percentages of GFP^+^ cells are depicted as in 3A. Note the significant increase in the percentage of GFP^+^ human cells in the spleen and maintenance of the percentage of GFP^+^ human cells in the bone marrow. There were 5 mice per group in this experiment.

### Knockdown of HPRT Provides Resistance to 6TG in Primary Human Hematopoietic Progenitor Cells

We isolated human hematopoietic progenitor cells (HPC) from cord blood and transduced them with either sh0 or sh491 constructs containing a GFP marker driven by the human PGK promoter (sh0G or sh491G). Cells were transduced with concentrated virus at an MOI of 1 to achieve transduction efficiency ranging from 20–30% as assessed by flow cytometry. In order to quantify HPRT expression levels in stably transduced HSCs, GFP^+^ cells were sorted, total RNA was isolated and relative expression levels were analyzed by reverse-transcription, real-time PCR. We observed 90% reduction in HPRT mRNA levels in cells transduced with sh491G compared to controls (**Figure 2a**). In order to determine if the hematopoietic progenitors would similarly be provided with resistance to 6TG, we cultured un-sorted, transduced cells in the presence of increasing amounts of 6TG. Cells were assessed for GFP expression, counted and replated at the same density every 72 h. While 6TG inhibited proliferation of both sh0G and sh491G transduced cells in a dose dependent fashion, the extent of inhibition of sh491G cells was significantly less than that of the control cells (**Figure 2b**). Over time, the percentage of sh0G transduced cells diminished with or without treatment with 6TG. However, with 6TG treatment there was a dose dependent, significant increase in the percentage of sh491G cells (**Figure 2c**). Importantly, both sh0G and sh491G transduced cells stopped proliferating in the presence of cisplatinum, indicating that MMR remained intact and that the effects observed were specific to 6TG (**Supplemental** **Figure 3d**) [26]. These data indicate that human hematopoietic progenitor cells can be provided with specific resistance to 6TG with lentiviral delivered knockdown of HPRT.

### Knockdown of HPRT Allows for in vivo Maintenance of Transduced Human Hematopoietic Progenitor Cells by Treatment with 6TG

We next tested whether HPRT knockdown can provide sufficient resistance to 6TG to allow for *in vivo* selection of transduced HPCs. CD34^+^ cells were transduced with sh0G or sh491G, and injected into sub-lethally irradiated NOD/SCID recipients. After allowing 3 weeks for human hematopoiesis to establish, mice were treated with 2 mg/kg 6TG in drinking water or left untreated (UT). Higher doses of 6TG were not tolerated well, even if the recipients were transplanted with sh491G human CD34^+^ cells (not shown). After six weeks of treatment, engraftment of human cells was evaluated in the spleen and bone marrow. Efficient engraftment was observed in mice transplanted either with sh0G or sh491G transduced HPCs, indicating that the transduction procedure did not significantly impair the repopulating potential of UCB CD34^+^ cells (not shown). In 6TG treated recipients of sh0G transduced HPCs, we observed a significant decrease in the percentages of transduced human cells in the spleen and bone marrow (**Figure 3a**; an example of the gating strategy for analysis of flow cytometry is provided in **Supplemental** **Figure 4**). This decrease was noted in the total leukocyte population, as well as B-lymphoid and myeloid sub-populations. (There were insufficient numbers of human myeloid cells in the spleens to evaluate.) The reasons for this specific decrease are not clear, but it may reflect exhaustion of transduced, committed HPC or a non-specific toxicity of the engagement of RNA-interference machinery. Nonetheless, in contrast, in 6TG treated recipients of sh491G transduced human HPC there was a significant increase in GFP expressing leukocytes and B-lymphocytes in the spleen, and the percentages of GFP^+^ leukocytes, and B lymphocyte and myeloid sub-populations was maintained with 6TG treatment in the bone marrow (**Figure 3b**), indicating protection from the toxic effects of 6TG, at least in the lymphoid population, by knockdown of HPRT.

**Figure 4:**
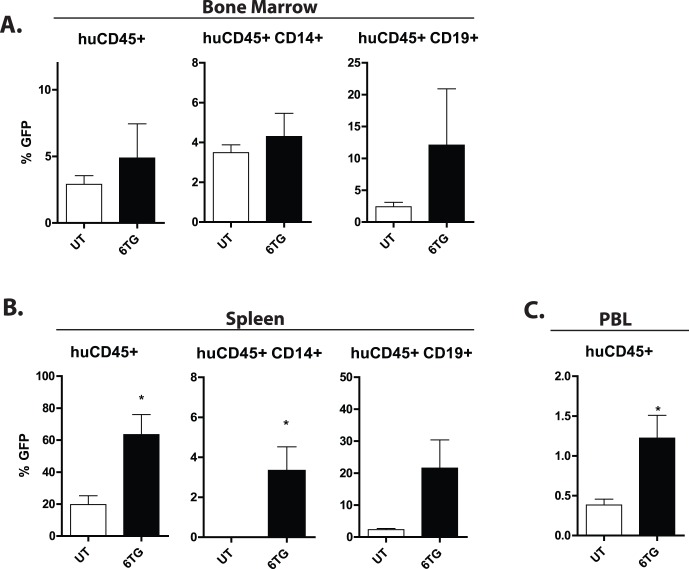
Knockdown of HPRT allows for maintenance of transduced HPC in NOD/SCID recipients. Human CD34^+^ umbilical cord blood cells were transduced with GFP expressing vectors with shRNA directed against HPRT, mixed with untransduced cells, and transplanted into sub-lethally irradiated NOD/SCID recipients. Mice were then treated with 6TG 2 mg/kg/d in the drinking water (n = 3) or left untreated (n = 4). After 6 weeks, bone marrow (A), spleen (B), and peripheral blood (C) were analyzed by flow cytometry for cell surface markers for total human leukocytes, B-lymphocyte and myeloid subpopulations, and GFP. A. Human HPC with reduced HPRT in the BM are maintained with or without treatment with 6TG. The percentages of GFP^+^ human leukocytes (huCD45^+^), and B-lymphocyte (huCD45^+^CD19^+^) and myeloid (huCD45^+^CD14^+^) sub-populations are depicted. While there is a trend for larger percentages of transduced cells in the 6TG treated recipients, the difference is not statistically significant. B. Human hematopoietic cells with reduced HPRT are selected with 6TG in the spleen. The percentages of GFP^+^ human leukocytes (huCD45^+^), and B-lymphocyte (huCD45^+^CD19^+^) and myeloid (huCD45^+^CD14^+^) sub-populations are depicted. The percentage of transduced human CD45^+^ and CD14^+^ cells was significantly greater in the 6TG treated recipients. C. Human hematopoietic cells with reduced HPRT are selected with 6TG in the peripheral blood. The percentages of GFP^+^ human leukocytes (huCD45^+^) are depicted. While the percentages of circulating human cells was low, there were significantly more transduced cells in 6TG treated recipients, as compared to untreated.

As we have never observed 6TG resistance in non-silencing control transduced mouse [26] or human cells, the non-silencing control vector was omitted from subsequent experiments to reduce the numbers of experimental mice. In an independent experiment with intentionally lower initial percentages of transduced cells, the percentage of transduced cells was maintained in the bone marrow of 6TG treated recipients (**Figure 4a**), similar to what we observed in the previous experiment,. We again observed a significant increase in sh491G transduced CD45^+^ human cells in the spleens of mice treated with 6TG (**Figure 4b**). While we did not detect GFP^+^ myeloid cells (CD14^+^) in the spleens of UT controls, we did detect transduced myeloid cells in the spleens of 6TG treated recipients. In addition, significant increases in the percentages of circulating GFP^+^ human cells in the peripheral blood were noted (**Figure 4c**). Taken together, these results indicate that knockdown of HPRT allows for multi-lineage maintenance of human HPCs in vivo.

### Maintenance of Cells with Reduced HPRT Activity Stems from Primitive HPC

**Figure 5:**
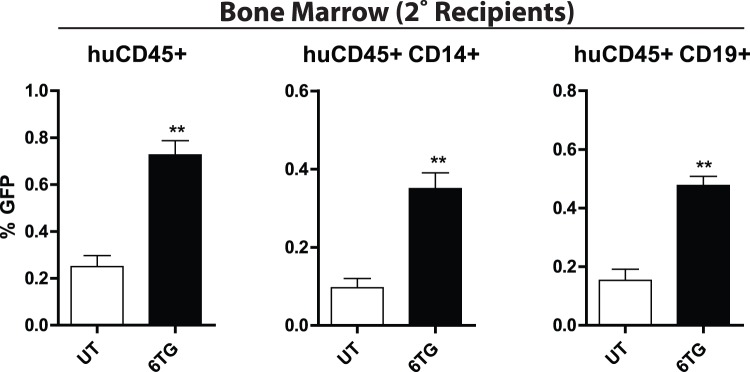
Knockdown of HPRT allows for selection from primitive HPC in vivo. Bone marrow cells from primary recipients of sh491G transduced human UCB cells were transplanted into sub-lethally irradiated secondary recipients. After 3 weeks, secondary recipients were left untreated (UT) or treated with 6TG. Six weeks later, tissues were harvested for analysis by flow cytometry. The percentages of GFP^+^ human leukocytes (huCD45^+^), and B-lymphocyte (huCD45^+^CD19^+^) and myeloid (huCD45^+^CD14^+^) sub-populations in the bone marrow are depicted. There were 3 mice per group in this experiment.

The life-span of myeloid cells is relatively short, and the presence of transduced myeloid progenitors, weeks after transplantation is indicative of successful transduction and selection from HPC. However, secondary transplantation is a more stringent measure of primitive HPC function. Thus, serial transplantations were performed to determine whether primitive HPC had indeed been transduced and that their contribution to multi-lineage hematopoiesis could be enhanced upon 6TG treatment. BM cells from untreated primary recipients of sh491G human UCB cells were injected into secondary sub-lethally irradiated mice. Three weeks later, secondary recipients were treated with 6TG or left untreated and hematopoietic cells were harvested for analysis 6 weeks later. Multi-lineage engraftment by human cells was observed in all mice in the BM as well as the spleen (not shown). A portion of human cells were GFP^+^, albeit at very low levels, indicating that very primitive HPCs had been transduced much less effectively than more committed progenitors. Enrichment of transduced human CD45^+^ cells was noted in the BM of 6TG treated mice compared to controls (**Figure 5**). This enrichment was not detected in the spleen, as the percentages of GFP^+^ human cells in this compartment were at or below the lower limit of detection (**Supplementary** **Figure 5**). The differentiated progeny (CD19^+^ and CD14^+^) also showed an increase in the BM compared to UT controls, indicating that the transduced HPCs maintained their proliferative and differentiation capacity and had a selective advantage with 6TG treatment.

## Discussion

In this study, we demonstrated the ability of 6TG to mediate *in vitro* selection and *in vivo* maintenance of genetically modified human HSCs expressing lentiviral delivered shRNAs targeting HPRT. The transduced HSCs retained their ability to self-renew, proliferate and differentiate into multiple lineages of the blood system, and were selectively able to survive in the presence of 6TG. Selection for transduced cells was significant in the spleens and peripheral blood of primary recipients. Importantly, though, drug resistance provided by knockdown of HPRT did not lead to dramatic increases in the percentages of transduced cells in the BM of primary recipients. Rather, percentages were maintained at levels similar to untreated recipients. The ability to repopulate and amplify in secondary recipients indicated that when selection did occur, it originated from transduced primitive long-term HPCs. Taken together, the extent of selection, particularly in the bone marrow, suggests that selection is most effective in more committed progenitor populations.

These results extend our previous report in which we showed efficient *in vivo* selection of murine HSCs transduced with LVs expressing HPRT shRNAs [26]. Recent studies by Hacke *et al* [29] used 6TG as a single agent for pre-transplant conditioning as well as *in vivo* selection of HPRT deficient mouse BM, corroborating the effectiveness of this strategy. Efficacy in human hematopoietic progenitor cells had not been previously demonstrated, though. Xenografted immunocompromised mice such as those described in this report are currently the most effective pre-clinical models with which to study human hematopoiesis. In our studies, selection for transduced hematopoietic cells was most pronounced in the spleens of primary recipients, although HPRT deficient cells were maintained in the BM of 6TG treated mice, while control transduced cells were differentially depleted. This may indicate that selection of transduced cells with 6TG resistance is most effective in more committed progenitor cells. Nonetheless, selection was detectable in the bone marrow of secondary recipients, indicating that drug resistance is propagated from the most primitive of hematopoietic progenitor cells to more committed cells, which may be selected.

Adverse events directly related to retroviral-mediated insertional mutagenesis in human trials, have been reported in some patients enrolled in gene-therapy clinical trials [5], [6], [30]. These effects are in part, mediated by the high MOIs used to achieve high transduction efficiencies. Therefore, our strategy was to transduce HPC at low MOIs in order to reduce the number of viral integrants per cell and subsequently enrich for the relatively few transduced HPCs. In addition, to further preserve the ‘stemness’ and self-renewal properties of HSCs, we transduced the cells immediately following isolation without growth factor pre-stimulation. As a result, transduction efficiencies of only 20–30% were achieved following two subsequent rounds of virus exposure using MOI of 1 for each transduction. While we were able to achieve increases of up to 80% of transduced human cells, the extent and consistency of selection that we observed was less than that of other reports with ΔMGMT [22]. Differences in experimental design, including the timepoints at which engraftment and selection efficiency were examined, may contribute to these differences.

In conclusion, our studies demonstrate that the HPRT-knockdown-6TG selection system can be used to increase the percentage of genetically modified human hematopoietic cells in experimental systems *in vitro* and *in vivo*. While this approach has several advantages, it may be limited to selection of more committed progenitor cells, rather than hematopoietic stem cells, perhaps limiting the clinical utility of this strategy. Whether optimized treatment conditions will enhance selection of HSC or if such a strategy may prove valuable in specific contexts will have to be determined with further work.

## Materials and Methods

### Ethics Statement

Human umbilical cord blood samples were collected after written informed consent of the donors by ClinImmune Labs with approval of the Colorado Multiple Institutional Review Board (IORG0000433). All animal experiments were approved by the Animal Care and Use Committee of the University of Colorado Denver.

### Isolation of Umbilical Cord Blood CD34^+^ Cells and Cell Culture

Human umbilical cord blood samples unsuitable for clinical use were obtained from the ClinImmune Labs or were purchased from AllCells (Emeryville, CA). CD34^+^ cells were isolated from cord blood using the CD34 Positive selection kit as per the manufacturers’ instructions (Stem Cell Technologies, Canada). Following magnetic bead separation, the viability of the isolated CD34^+^ cells was routinely 95%. Purity was determined by staining the cells with human CD34 antibody and analysis by flow cytometry. CD34^+^ cells were cultured in Iscove Modified Dulbecco Medium (IMDM) supplemented with 1% FBS and the following cytokines: stem cell factor (100 ng/ml), Flt3 ligand (100 ng/ml) and thrombopoietin (50 ng/ml) (SFT).

Molm13 and REH cell lines [31], [32], generous gifts from the laboratory of James DeGregori, were maintained in RPMI media supplemented with 10% FBS. Cells were seeded at 2×10^5^ cells/ml and after incubation in the absence or presence of 6TG live cell concentrations were determined by propidium iodide exclusion and flow cytometry.

### Lentiviral Transduction

Lentiviral constructs with shRNAs directed against murine and human HPRT in the pLKO.1 vector were purchased from Open Biosystems (Huntsville, AL). The non-silencing control in the pLKO.1 vector was purchased from Sigma Aldrich (St. Louis, MO). Constructs were modified to express GFP by cloning out the puromycin resistance gene using restriction endonucleases KpnI and BamHI, and PCR ligation of these restriction sites to the GFP gene from MSCV-iresGFP. Lentivirus was prepared by transient transfection of 293FT cells with transfer vectors along with third generation packaging constructs (pMDLg/pRRE+pRSV-Rev+pMD2.G). Viral titers and MOI were determined with serial dilution of virus containing media on NIH3T3 cells and flow cytometry for GFP. AML and ALL cell lines were transduced with unconcentrated virus supernatant overnight in the presence of 8 µg/ml polybrene and selected in puromycin (0.5 µg/ml). 5×10^5^ CD34^+^ cells were infected with concentrated viral stocks at a multiplicity of infection (MOI) of 1 on RetroNectin-coated plates (Takara, Japan) in the presence of proteasome inhibitor (MG132, 0.5 µM) and SFT in StemSpan H3000 media (Stem Cell Technologies, Canada). After two rounds of viral transduction, cells were maintained in IMDM media with SFT. Transduction efficiency was calculated by determining the percentage of GFP^+^ cells by flow cytometry.

### Reverse Transcription and Real-time PCR

Real-time PCR was performed in an ABI Prism 7900 (Applied Biosystems) with Taqman PCR using primers and probes from Roche (Universal ProbeLibrary Assay Design Center). Actin expression was used for normalization of gene expression.

### Mice

NOD/LtSz scid/scid (NOD/SCID) mice were obtained from Jackson Laboratories (Bar Harbor, ME) and maintained in the animal facility at the University of Colorado Denver. All mice were housed in sterile micro-isolators under standard conditions of care.

### Bone Marrow Transplantation and 6TG Treatment

Mice that were to receive transplants were irradiated at 6 to 8 weeks of age with 350 cGy total body irradiation administered from a ^137^ Cs source. Irradiated mice were treated with the enrofloxacin antiobiotic in drinking water (0.314 mg/ml) to prevent infectious complications. CD34^+^ cells isolated from cord blood were transduced overnight with LVs and transplanted (2×10^5^ cells/mouse) in 100 µl volume of phosphate-buffered saline (PBS) supplemented with 1% BSA via tail vein into sub-lethally irradiated female NOD/SCID primary recipients. Human cell engraftment in transplanted mice was confirmed in peripheral blood samples taken from the tail vein by flow cytometric analysis of CD45^+^ human cells, 3 weeks post transplantation. Thereafter, mice were left untreated or treated with 6TG in drinking water at a dose of approximately 2 mg/kg body weight, based on estimated water consumption of 5 ml/day and average weight of 20 g/mouse. This dose was the highest dose tolerated by NOD/SCID mice long term (not shown). All animals were euthanized after 6 weeks of treatment for assessment of numbers and types of human cells detectable in bone marrow and spleen. Secondary recipients were irradiated and transplanted with bone marrow from primary recipients at a ratio of 1∶4 (donor:recipient) and euthanized after 6 weeks.

### Flow Cytometric Analysis

All antibodies were purchased from eBiosciences (San Diego, CA). Peripheral blood collected from lateral tail vein was treated with hemolytic buffer and stained with antibodies directed against human CD45. Bone marrow (BM) cell suspensions were prepared from tibias and femurs and from spleens. Cells were hemolyzed and lineage analysis was performed by staining the cells with human antibodies against CD45, CD14, CD19 along with mouse CD45.1. After staining, cells were washed in FACS buffer and analyzed using Galios flow cytometer (Millipore, MA). At least 500,000 events were acquired for each sample and analyzed using Kaluza software (Beckman Coulter, CA). Transduced cells in each sub-population were determined by GFP expression.

### Statistics

Data are representative of three independent experiments. We used unpaired t-test or one-way ANOVA to determine statistical significance between 2 groups or multiple groups respectively. Bonferroni's multiple comparison test was used in conjunction with ANOVA. All of the statistical analyses were performed with GraphPad Prism 4 (GraphPad Software, San Diego, CA).

## Supporting Information

Figure S1 **Knockdown of HPRT confers resistance to 6TG in REH cells.** REH cells were transduced with constructs expressing shRNA directed against HPRT (49–53) and selected in puromycin or were left untransduced (UT). **A.** Whole cell lysates were analyzed by western blot with antibodies directed against HPRT and actin. **B.** Cells were treated for 72 hours with 6TG or were left untreated, and then counted by propidium iodide exclusion and flow cytometry. The number of live cells compared to Mock transduced, untreated cells is depicted. Note that cells transduced with construct 50 were not inhibited by 6TG at this dose.
(EPS)

Figure S2 **Knockdown of HPRT confers resistance to 6TG in human hematopoietic cells.** REH cells were transduced with lentiviral vectors expressing non-silencing control sequences (sh0 and sh0G) or shRNAs directed against HPRT (sh491 and sh50) and selected in puromycin. A & B. Construct 491 most effectively knocks down HPRT. The extent of knockdown of HPRT was measured by reverse-transcription, real-time PCR with primers specific for HPRT (A) and western blotting (B). Constructs 491 and 50 efficiently knocked down HPRT as compared to untransduced controls (*p<0.05). In these cells the extent of knockdown was not significantly greater with construct 491, as compared to construct 50. C. Construct 491 provides the greatest resistance to 6TG. Transduced cells were treated with increasing doses of 6TG, and the number of live cells was measured by flow cytometry and propidium iodide exclusion. Construct 491 provided the best resistance to 6TG.
(EPS)

Figure S3 **Knockdown of HPRT specifically protects cells from 6TG induced apoptosis.** A & B. Knockdown of HPRT abrogates the apoptotic effects of 6TG in human cell lines. Molm13 (A) or REH (B) cells were transduced with vectors expressing non-silencing control sequence (sh0) or shRNA directed against HPRT (sh491 and sh50) and were treated with 6TG at the indicated doses for 72 hours. Cells were then assessed for apoptosis by staining for annexin V and with propidium iodide using flow cytometry. The total percentage of early and late apoptotic (Annexin V^+^/PI^Neg^+Annexin V^+^/PI^+^) is depicted. C. Knockdown of HPRT is persistent over time. Molm13 cells were transduced with sh0 or sh491 and selected in puromycin. Assessment of HPRT expression and sensitivity to 6TG were assessed immediately after selection and after 4 weeks of proliferation (without puromycin selection). D. The protective effects of knockdown of HPRT are specific to 6TG. UCB cells were transduced with vector expressing GFP and a non-silencing shRNA (sh0G) or shRNA directed against HPRT (sh491G) and treated with cisplatinum at the indicated doses. In contrast to treatment with 6TG, in which the percentage of 491G transduced cells increases, the percentage of GFP^+^ cells decreases with cisplatinum, with either shRNA sequence.
(EPS)

Figure S4 **Flow cytometry gating scheme.** Bone marrow, spleen or peripheral blood cells were stained with antibodies directed against human CD45, CD19 and CD14 and analyzed by flow cytometry. Kaluza software was used to measure the percentage of GFP^+^ cells within specific sub-populations. An example of the gating schema with data from untreated and 6TG treated recipients is demonstrated.
(EPS)

Figure S5 **Low level engraftment of transduced human cells in the spleens of secondary recipients.** Bone marrow cells from primary recipients of 491G transduced human UCB cells were transplanted into sub-lethally irradiated secondary recipients. After 3 weeks, secondary recipients were left untreated (UT) or treated with 6TG. Six weeks later, tissues were harvested for analysis by flow cytometry. The percentages of GFP^+^ human leukocytes (huCD45^+^), and B-lymphocyte (huCD45^+^CD19^+^) and myeloid (huCD45^+^CD14^+^) sub-populations in the spleen are depicted. The lower limit of detection of GFP^+^ cells in these assays is approximately 0.05%.
(EPS)

Table S1 **shRNA construct identification and sequences.**
(PDF)

## References

[1] Aiuti A, Cattaneo F, Galimberti S, Benninghoff U, Cassani B, et al (2009) Gene therapy for immunodeficiency due to adenosine deaminase deficiency. N Engl J Med 360: 447–458. 19179314 10.1056/NEJMoa0805817

[2] Boztug K, Schmidt M, Schwarzer A, Banerjee PP, Diez IA, et al (2010) Stem-cell gene therapy for the Wiskott-Aldrich syndrome. N Engl J Med 363: 1918–1927. 21067383 10.1056/NEJMoa1003548 PMC3064520

[3] Cartier N, Hacein-Bey-Abina S, Bartholomae CC, Veres G, Schmidt M, et al (2009) Hematopoietic stem cell gene therapy with a lentiviral vector in X-linked adrenoleukodystrophy. Science 326: 818–823. 19892975 10.1126/science.1171242

[4] Hacein-Bey-Abina S, Le Deist F, Carlier F, Bouneaud C, Hue C, et al (2002) Sustained correction of X-linked severe combined immunodeficiency by ex vivo gene therapy. N Engl J Med 346: 1185–1193. 11961146 10.1056/NEJMoa012616

[5] Ott MG, Schmidt M, Schwarzwaelder K, Stein S, Siler U, et al (2006) Correction of X-linked chronic granulomatous disease by gene therapy, augmented by insertional activation of MDS1-EVI1, PRDM16 or SETBP1. Nat Med 12: 401–409. 16582916 10.1038/nm1393

[6] Hacein-Bey-Abina S, Von Kalle C, Schmidt M, McCormack MP, Wulffraat N, et al (2003) LMO2-associated clonal T cell proliferation in two patients after gene therapy for SCID-X1. Science 302: 415–419. 14564000 10.1126/science.1088547

[7] Modlich U, Bohne J, Schmidt M, von Kalle C, Knoss S, et al (2006) Cell-culture assays reveal the importance of retroviral vector design for insertional genotoxicity. Blood 108: 2545–2553. 16825499 10.1182/blood-2005-08-024976 PMC1895590

[8] Montini E, Cesana D, Schmidt M, Sanvito F, Ponzoni M, et al (2006) Hematopoietic stem cell gene transfer in a tumor-prone mouse model uncovers low genotoxicity of lentiviral vector integration. Nat Biotechnol 24: 687–696. 16732270 10.1038/nbt1216

[9] Levine BL, Humeau LM, Boyer J, MacGregor RR, Rebello T, et al (2006) Gene transfer in humans using a conditionally replicating lentiviral vector. Proc Natl Acad Sci U S A 103: 17372–17377. 17090675 10.1073/pnas.0608138103 PMC1635018

[10] Abonour R, Williams DA, Einhorn L, Hall KM, Chen J, et al (2000) Efficient retrovirus-mediated transfer of the multidrug resistance 1 gene into autologous human long-term repopulating hematopoietic stem cells. Nat Med 6: 652–658. 10835681 10.1038/76225

[11] Hesdorffer C, Ayello J, Ward M, Kaubisch A, Vahdat L, et al (1998) Phase I trial of retroviral-mediated transfer of the human MDR1 gene as marrow chemoprotection in patients undergoing high-dose chemotherapy and autologous stem-cell transplantation. J Clin Oncol 16: 165–172. 9440739 10.1200/JCO.1998.16.1.165

[12] Hildinger M, Fehse B, Hegewisch-Becker S, John J, Rafferty JR, et al (1998) Dominant selection of hematopoietic progenitor cells with retroviral MDR1 co-expression vectors. Hum Gene Ther 9: 33–42. 9458240 10.1089/hum.1998.9.1-33

[13] Sorrentino BP, Brandt SJ, Bodine D, Gottesman M, Pastan I, et al (1992) Selection of drug-resistant bone marrow cells in vivo after retroviral transfer of human MDR1. Science 257: 99–103. 1352414 10.1126/science.1352414

[14] Williams DA, Hsieh K, DeSilva A, Mulligan RC (1987) Protection of bone marrow transplant recipients from lethal doses of methotrexate by the generation of methotrexate-resistant bone marrow. J Exp Med 166: 210–218. 3298524 10.1084/jem.166.1.210 PMC2188648

[15] Warlick CA, Diers MD, Wagner JE, McIvor RS (2002) In vivo selection of antifolate-resistant transgenic hematopoietic stem cells in a murine bone marrow transplant model. J Pharmacol Exp Ther 300: 50–56. 11752096 10.1124/jpet.300.1.50

[16] Corey CA, DeSilva AD, Holland CA, Williams DA (1990) Serial transplantation of methotrexate-resistant bone marrow: protection of murine recipients from drug toxicity by progeny of transduced stem cells. Blood 75: 337–343. 1967217

[17] Cowan KH, Moscow JA, Huang H, Zujewski JA, O'Shaughnessy J, et al (1999) Paclitaxel chemotherapy after autologous stem-cell transplantation and engraftment of hematopoietic cells transduced with a retrovirus containing the multidrug resistance complementary DNA (MDR1) in metastatic breast cancer patients. Clin Cancer Res 5: 1619–1628. 10430060

[18] Maze R, Carney JP, Kelley MR, Glassner BJ, Williams DA, et al (1996) Increasing DNA repair methyltransferase levels via bone marrow stem cell transduction rescues mice from the toxic effects of 1,3-bis(2-chloroethyl)-1-nitrosourea, a chemotherapeutic alkylating agent. Proc Natl Acad Sci U S A 93: 206–210. 8552605 10.1073/pnas.93.1.206 PMC40207

[19] Beard BC, Trobridge GD, Ironside C, McCune JS, Adair JE, et al (2010) Efficient and stable MGMT-mediated selection of long-term repopulating stem cells in nonhuman primates. J Clin Invest 120: 2345–2354. 20551514 10.1172/JCI40767 PMC2898586

[20] Giordano FA, Sorg UR, Appelt JU, Lachmann N, Bleier S, et al (2011) Clonal inventory screens uncover monoclonality following serial transplantation of MGMT P140K-transduced stem cells and dose-intense chemotherapy. Hum Gene Ther 22: 697–710. 21319998 10.1089/hum.2010.088

[21] Davis BM, Koc ON, Gerson SL (2000) Limiting numbers of G156A O(6)-methylguanine-DNA methyltransferase-transduced marrow progenitors repopulate nonmyeloablated mice after drug selection. Blood 95: 3078–3084. 10807772

[22] Zielske SP, Reese JS, Lingas KT, Donze JR, Gerson SL (2003) In vivo selection of MGMT(P140K) lentivirus-transduced human NOD/SCID repopulating cells without pretransplant irradiation conditioning. J Clin Invest 112: 1561–1570. 14617757 10.1172/JCI17922 PMC259124

[23] Adair JE, Beard BC, Trobridge GD, Neff T, Rockhill JK, et al (2012) Extended survival of glioblastoma patients after chemoprotective HSC gene therapy. Sci Transl Med 4: 133ra157. 10.1126/scitranslmed.3003425 PMC3650895 22572881

[24] Erlichman C, Moor M (2001) Carcinogenesis: A late complication of cancer chemotherapy. In: Chabner BA, Longo, D.L., editor. Cancer Chemotherapy & Biotherapy. 3rd ed. Philadelphia: Lippincott Williams & Wilkens. 67–84.

[25] Pedersen-Bjergaard J, Andersen MK, Christiansen DH, Nerlov C (2002) Genetic pathways in therapy-related myelodysplasia and acute myeloid leukemia. Blood 99: 1909–1912. 11877259 10.1182/blood.v99.6.1909

[26] Porter CC, DeGregori J (2008) Interfering RNA-mediated purine analog resistance for in vitro and in vivo cell selection. Blood 112: 4466–4474. 18587011 10.1182/blood-2008-03-146571 PMC2597122

[27] Pui CH, Relling MV (2000) Topoisomerase II inhibitor-related acute myeloid leukaemia. Br J Haematol 109: 13–23. 10848777 10.1046/j.1365-2141.2000.01843.x

[28] Masunaga Y, Ohno K, Ogawa R, Hashiguchi M, Echizen H, et al (2007) Meta-analysis of risk of malignancy with immunosuppressive drugs in inflammatory bowel disease. Ann Pharmacother 41: 21–28. 17200426 10.1345/aph.1H219

[29] Hacke K, Szakmary A, Cuddihy AR, Rozengurt N, Lemp NA, et al.. (2012) Combined preconditioning and in vivo chemoselection with 6-thioguanine alone achieves highly efficient reconstitution of normal hematopoiesis with HPRT-deficient bone marrow. Exp Hematol 40: 3–13 e13. 10.1016/j.exphem.2011.09.009 PMC4305396 22001673

[30] Metais JY, Dunbar CE (2008) The MDS1-EVI1 gene complex as a retrovirus integration site: impact on behavior of hematopoietic cells and implications for gene therapy. Mol Ther 16: 439–449. 18227842 10.1038/sj.mt.6300372

[31] Rosenfeld C, Goutner A, Choquet C, Venuat AM, Kayibanda B, et al (1977) Phenotypic characterisation of a unique non-T, non-B acute lymphoblastic leukaemia cell line. Nature 267: 841–843. 197411 10.1038/267841a0

[32] Matsuo Y, MacLeod RA, Uphoff CC, Drexler HG, Nishizaki C, et al (1997) Two acute monocytic leukemia (AML-M5a) cell lines (MOLM-13 and MOLM-14) with interclonal phenotypic heterogeneity showing MLL-AF9 fusion resulting from an occult chromosome insertion, ins(11;9)(q23;p22p23). Leukemia 11: 1469–1477. 9305600 10.1038/sj.leu.2400768

