# Supplementary material for: Knockdown of HPRT for Selection of Genetically Modified Human Hematopoietic Progenitor Cells
Source: PLoS One. 2013 Mar 15;8(3):e59594. doi: 10.1371/journal.pone.0059594 (PMC3598703; doi:10.1371/journal.pone.0059594)
Supplement: Table S1 — shRNA construct identification and sequences. (PDF) [file pone.0059594.s006.pdf]

Supplemental Table 1. shRNA constructs

Construct Number	TRC Number	Sense strand sequence
49	TRCN0000035049	GCTATTGTAATGACCAGTCAA
50	TRCN0000035050	CCAGGTTATGACCTTGATTTA
51	TRCN0000035051	CCAGACTTTGTTGGATTTGAA
52	TRCN0000035052	CTAATCATTATGCTGAGGATT
53	TRCN0000035053	GTGTCATTAGTGAAACTGGAA
491	TRCN0000110491	GCACTGAATAGAAATAGTGAT
Non-silencing control	SHC002	CAACAAGATGAAGAGCACCAA
